# Endoscopic submucosal dissection for a soft-palate lesion with use of a small-bore tracheal tube combined with floss traction

**DOI:** 10.1055/a-2307-6132

**Published:** 2024-05-17

**Authors:** Sha Shi, Jing Ye, Qian Feng

**Affiliations:** 1 426111 Department of Gastroenterology, Liaocheng Peopleʼs Hospital, Liaocheng, China; 2 426111 Department of Pathology, Liaocheng Peopleʼs Hospital, Liaocheng, China

Endoscopic submucosal dissection (ESD) of the middle pharynx is rarely reported, with just one case previously reported by Chen et al. 1 . Several articles have reported that transoral forceps 2 and transnasal endoscopy 3 are convenient for pharyngeal ESD; however, the former method requires two endoscopes, and the ultrathin endoscope has a poor field of view, and small working-channel and scope diameters, which are not conducive to operation.

Here, we present a successful case of ESD performed on the soft palate ( Video 1 ). A 56-year-old man, with a history chemoradiotherapy for multiple synchronous advanced esophageal and hypopharyngeal squamous cell carcinomas, was found to have a lesion extending from the right soft palate to the oral side of the uvula and underwent follow-up endoscopy. The patient was a long-time heavy smoker and drinker. The lesion was a 2.5 × 2.0-cm superficial flat lesion (0-IIb). It appeared bloodshot and rough, with a clear boundary and turned brown on narrow-band imaging mode ( Fig. 1 ). Histopathologic examination identified the lesion as being high grade intraepithelial neoplasia (HGIN).

An area of intraepithelial neoplasia of the soft palate is marked by Lugolʼs iodine staining, and endoscopic submucosal dissection of the lesion is performed with use of a small-bore tracheal tube combined with floss traction.Video 1

**Fig. 1:**
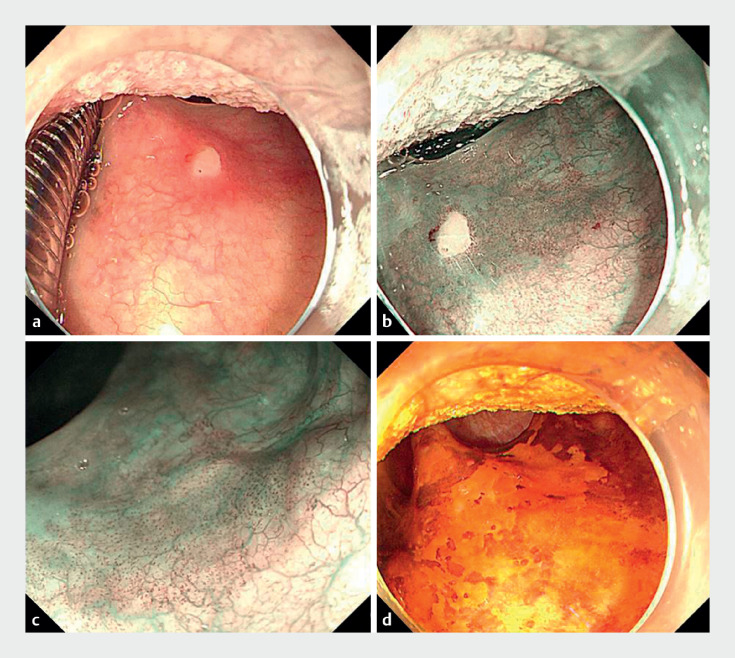
Endoscopic images of the lesion showing: **a** on white-light endoscopy, a 2.5 × 2.0-cm superficial flat lesion (0-IIb) that appeared bloodshot and rough, with a clear boundary; **b** the appearance on narrow-band imaging (NBI); **c** on magnified NBI, a type B1 intrapapillary capillary loop pattern; **d** on 0.75% Lugol chromoendoscopy, the clearly visible edge of the lesion.

An endotracheal tube with a 6.0-mm diameter was selected. The lesion was marked circumferentially after it had been stained with 0.75% Lugolʼs iodine, and a circumferential mucosal incision was made. A Sureclip (Micro-Tech Co., Ltd., Nanjing) with attached floss was placed to provide floss traction during ongoing dissection. The lesion was resected en bloc within 40 minutes, without any adverse events occurring ( Fig. 2 ). The uvula was protected to preserve its function. The patient’s postoperative pain was relieved by the use of lidocaine for 2 weeks. HGIN was confirmed pathologically in the excised specimen ( Fig. 3 ).

**Fig. 2:**
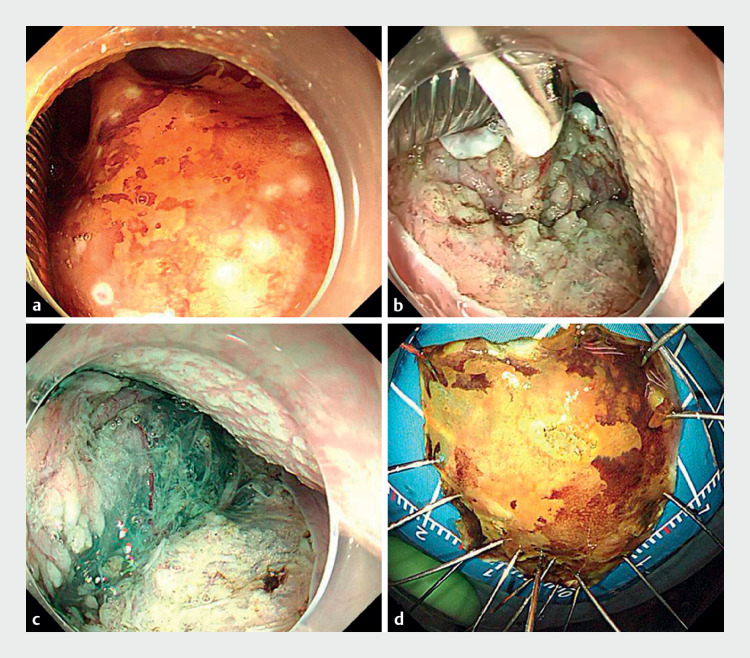
Images of the endoscopic submucosal dissection procedure with use of a small-bore tracheal tube combined with floss traction showing: **a** circumferential marking of the lesion; **b** circumferential incision of the lesion, with the help of a small-bore endotracheal tube and dental floss traction to continue submucosal dissection; **c** dissection of the subepithelial layer; **d** the macroscopic appearance of the specimen containing the lesion, which was resected en bloc with the assistance of floss traction.

**Fig. 3:**
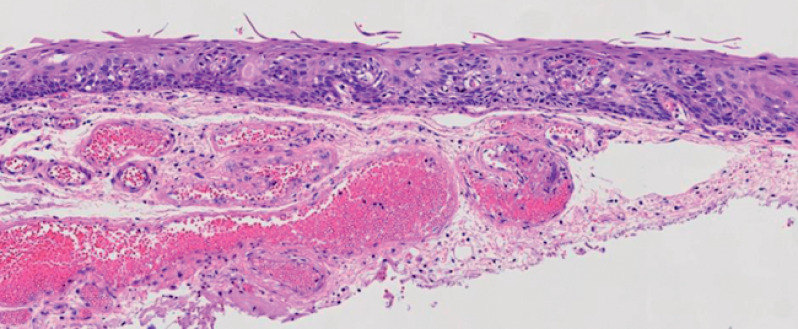
Histopathologic appearance of the specimen showing high grade squamous intraepithelial neoplasia (hematoxylin and eosin [H&E] stained; magnification × 200).

The middle pharynx is short and narrow, and surrounded by bony structures. The use of a small-bore tracheal tube combined with floss traction when performing ESD in this area is simple and convenient; it helped us to accurately identify the boundary of the lesion and improved the efficiency of dissection, in order to achieve en bloc resection. This technique has the potential to be a safe and effective treatment option for such cancers.

Endoscopy_UCTN_Code_TTT_1AO_2AG

## Notes

Endoscopy E-Videos https://eref.thieme.de/e-videos
*E-Videos* is an open access online section of the journal *Endoscopy* , reporting on interesting cases and new techniques in gastroenterological endoscopy. All papers include a high-quality video and are published with a Creative Commons CC-BY license. Endoscopy E-Videos qualify for HINARI discounts and waivers and eligibility is automatically checked during the submission process. We grant 100% waivers to articles whose corresponding authors are based in Group A countries and 50% waivers to those who are based in Group B countries as classified by Research4Life (see: https://www.research4life.org/access/eligibility/ ). This section has its own submission website at https://mc.manuscriptcentral.com/e-videos .
